# Sociodemographic and Clinical Predictors of Tuberculosis and Unsuccessful Treatment Outcomes in Davao City, Philippines: A Retrospective Cohort Study

**DOI:** 10.3390/ijerph22071154

**Published:** 2025-07-21

**Authors:** Alfredo A. Hinay, Marielle A. Mamalintaw, Joulei Mei L. Damasin, Bai Jana Shamera A. Dilangalen, Brent Adrian S. Montinola, Cristine Joy S. Napinas, Lester Evan Rey L. Valiente, Nathasia Lyn C. Insular, April Joy D. Parilla, Nelyn Mae T. Cadotdot, Nikka Mae R. Elipio, Jennifer Ashley H. Reyes, Avee Joy B. Dayaganon

**Affiliations:** 1 College of Medical and Biological Sciences, University of the Immaculate Conception, Davao City 8000, Philippinesninsular@uic.edu.ph (N.L.C.I.); aparilla@uic.edu.ph (A.J.D.P.); ncadotdot@uic.edu.ph (N.M.T.C.); nelipio@uic.edu.ph (N.M.R.E.); jreyes@uic.edu.ph (J.A.H.R.); adayaganon@uic.edu.ph (A.J.B.D.); 2 Graduate School Department, University of the Immaculate Conception, Davao City 8000, Philippines

**Keywords:** tuberculosis, risk factors, comorbidity, treatment outcomes, Davao City, Philippines

## Abstract

Background/Objectives: Tuberculosis (TB) remains a major public health challenge in Davao City, Philippines, with persistent issues in both disease burden and treatment outcomes. Understanding the risk factors for TB and its unsuccessful treatment is essential for guiding effective interventions. This study aimed to evaluate the association of sociodemographic and clinical factors with TB occurrence and to identify predictors of unsuccessful TB treatment outcomes among patients in Davao City. Methods: A retrospective cohort study was conducted using data from 521 patients diagnosed with drug-susceptible TB at Davao Chest Center between January 2021 and May 2024. The sociodemographic and clinical profiles of the patients were described using descriptive statistics. Chi-square tests were used to assess the associations between sociodemographic and clinical variables with TB risk and treatment outcomes. Results: The patient cohort was predominantly aged 31–50 years (n = 201, 38.58%), male (n = 284, 54.51%), and married (n = 285, 54.70%), with most residing in Districts I and II (n = 98, 38% each), and had no previous TB treatment (n = 344, 66.03%). Among the 456 patients assessed for comorbidities, 56.14% (n = 256) had at least one comorbidity. Evaluation of the risk factors for TB occurrence among the study population revealed that comorbidity status was not significantly associated with an increased risk of TB diagnosis (*p* = 0.682). However, among patients diagnosed with TB, the presence of comorbidities was significantly associated with unsuccessful treatment outcomes (*p* = 0.003). Conclusions: Although sociodemographic factors did not significantly influence TB risk or treatment outcomes, the presence of comorbidities was a significant predictor of unsuccessful TB treatment. These findings highlight the importance of integrating comorbidity management with TB care to improve treatment success in high-burden urban settings.

## 1. Introduction

Tuberculosis (TB) remains a critical global health challenge, ranking as the leading cause of death from a single infectious disease, accounting for 10.8 million new cases and 1.25 million deaths worldwide in 2023 [1,2,3]. The burden is particularly concentrated in Asia, with the Philippines among the top five countries contributing to more than half of global TB cases [4]. In 2023, the Philippines reported a TB incidence rate of 643 per 100,000 people, which is substantially higher than the Western Pacific regional average of 97 per 100,000 and 37,000 TB-related deaths, making TB one of the country’s top causes of mortality.

Despite the implementation of the WHO-endorsed Directly Observed Treatment Short-course (DOTS) strategy, the Philippines has experienced a decline in treatment success rates, dropping from 91% in 2017 to 76% in 2020, which is well below the global average of 86%. Several factors may explain this concerning decline. Most notably, the COVID-19 pandemic severely disrupted TB services, as health resources and personnel were redirected to the pandemic response, limiting access to diagnosis, treatment, and follow-up for patients with TB. Quarantine measures and movement restrictions further hindered patients’ ability to attend health facilities, resulting in missed doses and treatment interruptions [5]. Additionally, patient-related barriers, such as economic hardship, stigma, and limited understanding of the importance of treatment adherence, contribute to non-completion of therapy [6]. Health system challenges, including insufficient human resources, shortages of funds for patient support, and gaps in healthcare provider training, especially regarding drug-resistant TB, also played a role [6,7]. This decline is particularly alarming, as incomplete treatment is closely linked to the emergence of drug-resistant TB (DR-TB), which presents significant challenges owing to its complex and costly treatment regimens. Regional disparities in TB burden are evident, with Calabarzon reporting the highest notification rate at 40,467 cases, while Davao Region (Region XI) recorded 18,000 cases [8], underscoring the need for localized interventions.

Importantly, even among drug-susceptible TB (DSTB) cases, a substantial proportion of patients in the Philippines and across Asia experience unsuccessful treatment outcomes driven by factors such as treatment interruption, loss to follow-up, and death [2,9,10]. The interplay between sociodemographic factors, including age, sex, social stigma, and clinical profiles, such as prior TB treatment history and comorbidities (notably diabetes and HIV), further complicates treatment efforts, often resulting in poorer outcomes [11,12].

Given these multifaceted barriers, this study sought to investigate the correlation between sociodemographic profiles, comorbidities, and TB treatment outcomes in Davao City, Philippines. By analyzing data from TB treatment facilities within the city, this study aimed to provide a nuanced understanding of the local determinants influencing treatment success and failure. Although the study is rooted in the context of Davao City, the city’s diverse sociodemographic composition and the prevalence of comorbidities such as diabetes, hypertension, and HIV among patients reflect patterns observed in many other regions of the Philippines. The interplay between TB treatment outcomes and these comorbidities is a critical issue nationwide, suggesting that the findings and recommendations of this study can inform broader TB control strategies and policy development across the country. Identifying specific factors associated with poor outcomes could inform targeted interventions such as enhanced patient education and the integration of comorbidity management into TB care. Furthermore, the study’s methodology and analytical approach can serve as a model for similar investigations in other urban centers, strengthening the evidence base for context-specific interventions while supporting the refinement of the national TB program. These findings may support the development of more effective context-specific TB control programs and guide local health authorities in prioritizing resources, strengthening monitoring, and tailoring support for high-risk groups in the future. Ultimately, this approach will contribute to ongoing efforts to reduce the TB burden in Davao City and across the Philippines, providing practical insights for refining national TB strategies in line with global best practices.

## 2. Materials and Methods

### 2.1. Research Design

We conducted a retrospective cohort study using medical records from Davao Chest Center, the principal TB DOTS facility in Davao City. This study focused on individuals diagnosed and treated for TB between 1 January 2021 and 31 May 2024. Data were extracted from the patient registration forms and treatment logbooks to capture sociodemographic characteristics, clinical profiles, and TB treatment outcomes. This study adhered to the STROBE guidelines for observational studies.

### 2.2. Study Population and Data Source

The Davao Chest Center, located at 456 Villa Abrille St, Poblacion District, Davao City, serves as the primary TB DOTS facility in the region, making it the optimal setting for this investigation. The center’s strict adherence to the Philippine National Tuberculosis Control Program (NTCP) and the World Health Organization’s DOTS guidelines ensures standardized data collection, minimizes variability, and maintains high-quality medical records. The facility’s extensive patient database contains detailed sociodemographic and clinical information relevant to the multifactorial determinants of TB treatment outcomes in Davao City, Philippines.

### 2.3. Participants

#### 2.3.1. Eligibility Criteria

1. Clinical and bacteriological diagnosis of drug-susceptible TB at Davao Chest Center
2. Complete registration forms and treatment logbooks for the study period
3. Completed treatment course residency in Davao City

#### 2.3.2. Exclusion Criteria

1. Ongoing treatment at the time of data collection
2. Referral to other healthcare facilities
3. Incomplete registration forms
4. Missing information in treatment logbooks

### 2.4. Participant Selection

**Figure 1:**
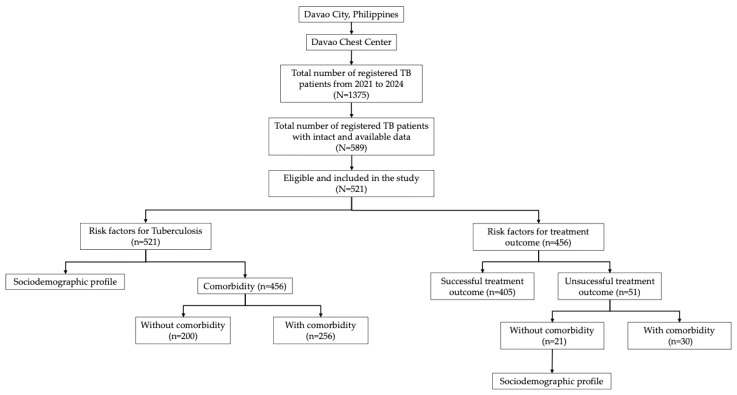
Flow diagram of study participants in Davao City, Philippines, 2021–2024 cohort (*n* = 521).

Figure 1 presents the flow diagram for patient selection and analysis. A total of 1375 TB patients were registered from 2021 to 2024, but only 589 had intact and available data. A large proportion was excluded primarily due to incomplete registration forms or missing information in the treatment logbooks. This was particularly pronounced during the COVID-19 pandemic, which affected the routine documentation. After applying eligibility criteria, 521 patients were included in the final cohort. For the primary analyses, 456 patients were assessed after excluding those who refused to provide their HIV-1 status. This study aimed to identify the risk factors of TB and unsuccessful treatment outcomes. For the analysis of TB risk factors, patients were further categorized by sociodemographic profile and comorbidity status, resulting in 200 patients without comorbidities and 256 patients with comorbidities. In the analysis of treatment outcomes, 405 patients achieved successful outcomes, while 51 experienced unsuccessful outcomes. Among those with unsuccessful outcomes, 21 had no comorbidities, and 30 had comorbidities. To accurately assess the true risk factors for unsuccessful treatment outcomes using sociodemographic profiles, this analysis focused specifically on patients without known comorbidities. This structured approach enabled a comprehensive evaluation of both the disease risk factors and determinants of treatment success within the study population.

### 2.5. Variables

#### 2.5.1. Exposures

Sociodemographic characteristics included age, sex, marital status, smoking status, residency (Congressional District), and previous TB treatment history.

Clinical characteristics comprised comorbidities (diabetes, hypertension, and HIV status) as recorded in the medical records. Nutritional status and other comorbidities were not included as they were not consistently recorded.

#### 2.5.2. Outcomes

The primary measure of interest was TB treatment outcomes, classified according to the NTCP/WHO guidelines. Successful treatment outcomes included patients who were either cured, defined as those with bacteriologically confirmed TB who were smear- or culture-negative in the last month of treatment and on at least one previous occasion, or who completed treatment without evidence of failure, even if bacteriological results were unavailable. Unsuccessful outcomes included treatment failure (remaining smear-positive at five months or later during treatment), death from any cause during treatment, and loss to follow-up (treatment interrupted for two consecutive months or more).

### 2.6. Data Collection

Data were extracted from the patient registration forms and treatment logbooks to ensure comprehensive clinical and sociodemographic information for each participant. This methodological approach allowed for a robust analysis of the relationship between patient characteristics and treatment outcomes among drug-susceptible TB patients in Davao City during the specified timeframe, while addressing potential limitations through strict inclusion and exclusion criteria.

### 2.7. Data Analysis

Data analysis used descriptive statistics for demographic characterization, chi-square tests to assess associations among categorical variables, and binary logistic regression to identify predictors of treatment outcomes.

## 3. Results

### 3.1. Sociodemographic Profile of Tuberculosis Patients

The sociodemographic data (Table 1) of 521 patients with TB at the Davao Chest Center included age, sex, marital status, smoking status, residency (Congressional District), and previous TB treatment history. Most patients were aged between 31 and 50 years (38.58%), with those aged 18–30 and over 50 years representing 34.36% and 27.06%, respectively. Males comprised 54.51% of the patient population, and married individuals accounted for 54.70% of the patient population. Smokers constituted 51.63% of the patients, and 66.03% had no previous history of TB treatment. Most patients resided in Districts I and II (38% each). These sociodemographic characteristics provide an important context for understanding the distribution of TB cases within the urban population of Davao City.

Table 2 presents the sociodemographic profile as a possible risk factor for unsuccessful tuberculosis treatment outcomes among 21 patients without known comorbidities in Davao City, Philippines, from 2021 to 2024. The analysis shows that none of the sociodemographic factors, including age group (*p* = 0.521), sex (*p* = 0.178), marital status (*p* = 0.226), smoking status (*p* = 0.582), previous treatment history (*p* = 0.861), and congressional district (χ^2^ = 2.81, *p* = 0.093), were statistically significant risk factors for unsuccessful TB treatment outcomes in this cohort, as all *p*-values were above the conventional threshold of 0.05. This suggests that within this specific group of patients without comorbidities, sociodemographic characteristics did not significantly influence the likelihood of an unsuccessful TB treatment outcome.

### 3.2. Comorbidity of Tuberculosis Patients

In this study, comorbidities were defined as the presence of diabetes mellitus, hypertension, or HIV infection, as documented in the patients’ medical records. Table 3 presents the distribution of comorbidity status among 456 individuals with tuberculosis in Davao City, Philippines, between 2021 and 2024. Among these patients, 43.86% (*n* = 200) had no comorbidities, whereas 56.14% (*n* = 256) had at least one comorbidity. The chi-square test revealed no significant association between comorbidity status and tuberculosis (*p* = 0.682), indicating that the presence of these comorbidities was not a significant risk factor for tuberculosis in this cohort during the study period.

Although Table 3 shows that comorbidity status was not significantly associated with the risk of developing tuberculosis in the Davao City cohort from 2021 to 2024, Table 4 reveals a different pattern when treatment outcomes were considered. Specifically, patients with comorbidities were significantly more likely to experience unsuccessful TB treatment outcomes than those without comorbidities (*p* = 0.003). This contrast highlights that, while comorbidities may not increase the likelihood of acquiring TB, they play a critical role in influencing the success of treatment, with comorbid patients facing a higher risk of poor outcomes.

## 4. Discussion

The findings from this retrospective cohort study in Davao City highlight the nuanced relationship between sociodemographic factors, comorbidity status, and tuberculosis (TB) treatment outcomes. Notably, sociodemographic variables, such as age, sex, marital status, smoking, previous treatment, and geographic district, did not show statistically significant associations with unsuccessful TB treatment outcomes among patients without comorbidities. This aligns with recent research indicating that, while sociodemographic characteristics can influence TB risk and access to care, their direct effect on treatment outcomes may be limited or context-dependent, especially in settings where healthcare access is relatively uniform [13,14,15].

In contrast, comorbidities were a significant risk factor for poor TB treatment outcomes in this cohort. Patients with comorbid conditions were more likely to experience unsuccessful treatment, although comorbidity status was not associated with the risk of acquiring TB. This finding is consistent with broader literature, which demonstrates that comorbidities such as diabetes, HIV, and chronic respiratory diseases can complicate TB management by impairing the immune response, increasing the risk of drug interactions, and making adherence to lengthy treatment regimens more challenging [16,17,18,19]. Notably, some studies have found that although comorbidities do not always increase TB incidence, they are consistently linked to higher rates of treatment failure and mortality [20,21,22,23].

Several measures were implemented to minimize potential sources of bias in this observational study. Selection bias was addressed by applying strict inclusion and exclusion criteria, ensuring that only patients with complete registration forms and treatment logbooks were included in the study. This approach helped improve the accuracy and reliability of the data, although we recognize that some degree of bias may remain due to the exclusion of incomplete records. To reduce information bias, data were extracted systematically from standardized medical records maintained at the Davao Chest Center, which adheres to the Philippine National Tuberculosis Control Program and WHO’s DOTS guidelines. This standardized data collection process aimed to enhance consistency and minimize variability in the recording of sociodemographic and clinical variables of interest. Although confounding cannot be entirely eliminated in observational research, we attempted to account for key sociodemographic and clinical factors in our analyses. Nevertheless, we acknowledge that unmeasured confounders, such as socioeconomic status and treatment adherence, could still influence the observed associations. By clearly defining the eligibility criteria, utilizing standardized data sources, and considering relevant covariates, we sought to reduce the impact of bias and confounding factors on our findings.

These results underscore the importance of integrating comorbidity screening and management into TB control programmes. Sociodemographic factors should not be overlooked, given their potential to influence health behaviors and healthcare access. The data suggest that targeted interventions for patients with comorbidities are likely to yield the greatest improvements in treatment outcomes. This approach is particularly relevant in urban settings, such as Davao City, where healthcare infrastructure may mitigate some sociodemographic disparities, but where the burden of chronic disease remains high. Future research should further delineate the specific comorbidities that most adversely affect TB treatment and evaluate the effectiveness of integrated care models in improving patient prognosis. The study reinforces that while sociodemographic factors provide an important context, comorbidities are a more direct and actionable determinant of TB treatment success in this population. Tailored interventions addressing comorbid conditions are essential to reduce unsuccessful outcomes and advance TB control efforts in high-burden settings.

This study had several limitations. First, the study period overlapped with the COVID-19 pandemic, which caused substantial disruptions in TB service delivery and may have affected both treatment outcomes and the completeness or accuracy of the medical records. Second, while our analysis showed that the presence of comorbidities was significantly associated with unsuccessful TB treatment outcomes, the limited number of respondents in each comorbidity subgroup prevented us from conducting further analyses to determine which specific types of comorbidities contributed most to these outcomes. Third, although HIV status was included as a comorbidity, data on whether HIV-positive patients were receiving antiretroviral therapy or had achieved viral suppression were unavailable, limiting our ability to assess the full impact of HIV management on TB outcomes. These factors should be considered when interpreting our findings, and future research should aim to collect more detailed clinical data, explore the individual effects of specific comorbidities, and account for the broader health system impact of public health emergencies.

## 5. Conclusions

This study highlights that comorbidities are a significant predictor of unsuccessful tuberculosis treatment outcomes in Davao City, whereas sociodemographic factors such as age, sex, and marital status do not appear to have a substantial impact. These findings emphasize the importance of prioritizing the identification and management of comorbid conditions in TB patients to improve treatment success rates. Integrating comorbidity screening and tailored interventions into TB control programs can help to address this critical risk factor. Strengthening these approaches is essential to reduce treatment failures and advance TB control efforts in the region.

## Figures and Tables

**Table 1:** Sociodemographic profile as possible risk factors of tuberculosis in Davao City, Philippines, 2021–2024 cohort (*N* = 521).

Sociodemographic Profile	n (%)	X^2^	df	*p*
Age				
18–30 years old	179 (34.36)	0.147	2	0.929
31–50 years old	201 (38.58)			
>50 years old	141 (27.06)			
Sex				
Male	284 (54.51)	0.425	1	0.514
Female	237 (45.49)			
Marital Status				
Single	189 (36.28)	0.780	3	0.854
Married	285 (54.70)			
Widowed	44 (8.45)			
Divorced	3 (0.58)			
Smoking				
No	252 (48.37)	0.160	1	0.689
Yes	269 (51.63)			
Previous Treatment No Yes	344 (66.03) 177 (33.93)	0.637	1	0.425
Congressional District				
I	198 (38)	0.500	2	0.779
II	198 (38)			
III	124 (23.80)			

**Table 2:** Sociodemographic profile as possible risk factors for unsuccessful treatment outcome for tuberculosis among patients without known comorbidities in Davao City, Philippines, 2021–2024 cohort (*N* = 21).

Sociodemographic Profile	% (*n*)	X^2^	df	*p*
Age				
18–30 years old	(8)	0.41	1	0.521
31–50 years old	(6)			
>50 years old	(7)			
Sex				
Male	(8)	1.81	1	0.178
Female	(13)			
Marital Status				
Single	(6)	1.47	1	0.226
Married	(13)			
Widowed	(2)			
Divorced	(0)			
Smoking				
No	(5)	0.30	1	0.582
Yes	(16)			
Previous Treatment No Yes	(13) (8)	0.03	1	0.861
Congressional District				
I	(8)	2.81	1	0.093
II	(9)			
III	(4)			

**Table 3:** Comorbidity as a risk factor of tuberculosis in Davao City, Philippines, 2021–2024 cohort (*N* = 456).

Comorbidity Status	% (*n*)	X^2^	df	*p*
Without comorbidity	43.86 (200)			
With comorbidity	56.14 (256)	0.884	1	0.682

**Table 4:** Comorbidity as possible risk factor of unsuccessful treatment outcome of tuberculosis in Davao City, Philippines, 2021–2024 cohort (*N* = 51, 11.18%).

Comorbidity Status	% (*n*)	X^2^	df	*p*
Without comorbidity	41.18 (21)	9.01	1	0.003
With comorbidity	58.82 (30)			

## Data Availability

The dataset is available on request from the authors.
